# Hepatic steatosis, detected by hepatorenal index in ultrasonography, as a predictor of insulin resistance in obese subjects

**DOI:** 10.1186/s40608-016-0118-0

**Published:** 2016-09-20

**Authors:** Victoria T. Isaksen, Maria A. Larsen, Rasmus Goll, Jon R. Florholmen, Eyvind J. Paulssen

**Affiliations:** 1 Research group of Gastroenterology and Nutrition, Department of Clinical Medicine, UiT The Arctic University of Norway, Tromsø, Norway; 2 Department of Gastroenterology, University Hospital of North Norway, Tromsø, Norway; 3 Victoria Therese Isaksen, Faculty of Health Sciences, UiT The Arctic University of Tromsø, P. O. Box 6050 Langnes, N-9037 Tromsø, Norway

**Keywords:** Body mass index, Hepatorenal index, HOMA1-IR, Insulin resistance, Liver steatosis, Metabolic syndrome, Morbid obesity, Non-alcoholic fatty liver disease, Quantification, Ultrasonography

## Abstract

**Background:**

The metabolic syndrome is a worldwide health issue, with non-alcoholic fatty liver disease (liver steatosis) being one of its features, particularly closely related to insulin resistance. This study aims to investigate whether quantification of hepatic steatosis by abdominal ultrasonography, using hepatorenal index, is a feasible tool for the prediction of insulin resistance, and thus the metabolic syndrome.

**Methods:**

Patients were recruited from the Centre of Obesity at the University Hospital of North Norway, and among participants from the Sixth Tromsø Study. Homeostasis Model Assessment of Insulin Resistance (HOMA1-IR) was measured, body mass index (BMI, kg/m^2^) calculated, and hepatorenal index (HRI), i.e. the ratio of liver to kidney optical densities, was measured by transabdominal ultrasonography. Receiver operating characteristic (ROC) analyses were performed, detecting insulin resistance at HOMA1-IR cut-off values >2.3 and >2.5.

**Results:**

Ninety participants were included in the study, of which 46 (51 %) had BMI ≥30 and 27 (30 %) had BMI ≥35. Overall, HRI at level 1.17 had sensitivity 0.90 and specificity 0.70 for predicting insulin resistance (HOMA1-IR >2.3) in all participants. For participants with BMI ≥30, HRI at level 1.17 had sensitivity 0.94 and specificity 0.70, and for BMI ≥35, HRI at level 1.17 had sensitivity 0.93 and specificity 0.75 for predicting HOMA1-IR >2.3. Setting the HRI limit at 1.42 gave low sensitivities and high specificities in all BMI groups. In the analysis predicting HOMA1-IR >2.5, sensitivity values were almost the same as in the analysis predicting HOMA1-IR >2.3, but specificity values were lower in this analysis.

**Conclusion:**

Detection and quantification of hepatic steatosis by ultrasound and the hepatorenal index is a feasible screening tool for identifying patients with low risk of having insulin resistance (IR, HRI <1.17), patients at risk of having IR (HRI 1.17-1.41) and patients with likely IR (HRI ≥1.42), especially in obese individuals.

## Background

Obesity is a major worldwide health issue due to its association with the metabolic syndrome (MetS). In 2013, 36.9 % of men and 38.0 % of women in developed countries were overweight or obese [1]. Components of MetS are increased waist circumference, hypertension, insulin resistance (IR), type 2 diabetes and dyslipidemia, i.e. hypertriglyceridemia and low levels of high density lipoproteins (HDL) [2, 3].

Non-alcoholic fatty liver disease (NAFLD) is closely associated with both IR and MetS, as patients with IR run a high risk of also having NAFLD, and NAFLD is a predictor of the metabolic disturbances associated with MetS [4–7].

Accordingly, components of the metabolic syndrome are also strongly associated to NAFLD in normal-weight subjects without diabetes, and in obese subjects diagnosed with NAFLD the prevalence have been found to be 67–71 % [8, 9]. Similarly, in subjects with diabetes mellitus type 2 or impaired glucose tolerance, the prevalence of NAFLD is found to be 30–70 % [10–12].

The association with high body weight makes NAFLD the most common type of liver disease in the developed world today, with a prevalence of approximately 30 % [13, 14], a number that is expected to increase [15].

The hepatic lipid metabolism is vulnerable to metabolic dysfunction, resulting in the accumulation of lipid droplets in the hepatocyte. The ‘two-hit theory’ model by Day [16] describes the pathogenesis of NAFLD. The ‘first hit’ is a hepatocellular lipid accumulation due to an imbalance of lipid uptake and combustion. The progression to the ‘second hit’ is defined as a hepatocellular steatohepatitis (Non- alcoholic steatohepatitis (NASH)), due to the imbalance between pro- and anti-inflammatory factors [16]. Among these, adipocytokines play a central role in this pathogenesis [17].

Insulin resistance is considered to be the most important pathophysiological mechanism of MetS [18, 19]. IR is characterized by impaired lowering of blood glucose through reduced glucose uptake in muscles, and lack of insulin effect on endogenous glucose production in liver. IR is also characterized by impaired insulin effect on lipid and protein metabolism, as well as impaired effect on a number of other organs [18, 20].

A common and efficient way of assessing insulin resistance is by the Homeostasis Model Assessment of insulin resistance (HOMA1-IR) [21, 22]. This model is based on measurements of fasting blood glucose and fasting insulin only. It has been proven to be equally good as the gold standard for measuring IR, i.e. the euglycemic clamp, in addition to being a more convenient test to perform. [23] A cut-off value of HOMA1-IR >2.3 has previously been shown to have a sensitivity of 76.8 % and a specificity of 66.7 % for identifying metabolic syndrome, whereas a cut-off value of 2.7 had the same test statistics for detecting insulin resistance [24].

Measurements of increased levels of alanine aminotransferase (ALT), aspartate aminotransferase (AST) and gamma-glutamyl transferase (γ-GT) may be used as an indicator of NAFLD when biopsy is contraindicated or not available, in combination with radiological methods, most often transabdominal ultrasonography (US) [25].

Subjective assessments of steatosis by US display a relatively large inter- and intra-observer variability. A way of reducing this variability is by measuring the liver and kidneys’ echogenicity on a histogram grayscale, and using the mean values for computing the hepatorenal index (HRI) [26]. HRI is a tool of quantifying the steatosis that is more reliable than subjective assessment alone. In a normal liver, HRI is in the range from 1.00 to 1.04. Hepatic steatosis is classified as mild (HRI 1.05–1.24), moderate (1.25–1.64) or severe (≥1.65) [26].

The availability and cost of abdominal US examinations and biochemical analyses such as that of insulin by ELISA will differ both between and within countries. In our department, ultrasonography examinations are easily accessible, whereas insulin measurements are not done on a routine basis, making use of US in the assessment of obese patients a practical approach.

As far as we know, the use of liver steatosis, assessed by HRI at ultrasonography as a predictor and screening method for IR, has not been examined in previous studies. Therefore, we aimed to investigate the test properties of this method.

### Aim of study

To examine whether hepatic steatosis, quantified by HRI, is a feasible test for detecting IR.

## Methods

Participants eligible for the study were adults with either obesity or elevated liver enzymes. Only individuals meeting the inclusion criteria were invited to participate in the study, and all those who accepted the invitation were included.

**Fig. 1:**
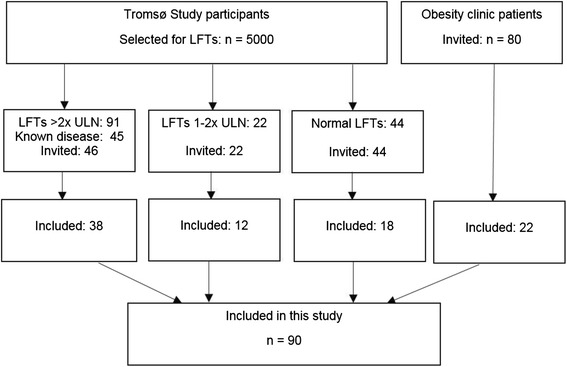
Flowchart of included participants from the Sixth Tromsø Study and the University Hospital Obesity Clinic. LFT: Liver function tests (i.e. liver enzymes), ULN: Upper limit of normal

Obese patients were recruited from the Centre of Obesity, Department of Gastroenterology at the University Hospital of North Norway (*n* = 80). Subjects with elevated liver enzymes were recruited from the Sixth Tromsø Study population (Fig. 1).

The Sixth Tromsø Study in 2008 is previously described [27]. Participants with values of AST, ALT or γ-GT above the Upper Limit of Normal (ULN) when examined in The Tromsø Study (n = 68), were invited to participate in our study. All participants who accepted the invitation were included in the study, and divided into two groups: liver enzymes 1–2× ULN and liver enzymes >2× ULN.

We also invited a control group with normal liver enzymes (*n* = 44), drawn from the main study population of the sixth Tromsø Study. This control group was sized and adjusted for sex and age to match the group of participants with liver enzymes >2× ULN. Participants who accepted the invitation were included in the study.

All participants signed a written consent, which included permission to use their data for follow-up studies. The Regional Committee of Medical Ethics of North Norway approved the study performed at the Centre of Obesity, including the approval of a biobank.

The Tromsø Study organisation approved access to data and participants for the follow-up study for the Sixth Tromsø Study. The main ethical approval given for the Sixth Tromsø Study by the Regional Committee of Medical Ethics of North Norway also covered the follow-up study.

Inclusion criteria for the subjects recruited from the Centre of Obesity were BMI >30 kg/m^2^ and age >18 years. Exclusion criteria were medically treated diabetes mellitus, severe heart disease or severe kidney failure. Patients that met the inclusion criteria at the Centre of Obesity were invited to participate in the study on their first consultation, or at their first group seminar during their treatment period. All participants recruited from the Centre of Obesity underwent an Oral Glucose Tolerance Test (OGTT), where the participants ingested 75 g of glucose dissolved in water. Fasting and postprandial blood glucose (mmol/L) and serum insulin (μmol/L) levels were measured at 30-min intervals for up to 180 min, and HOMA1-IR and WBISI were calculated as follows:

$$ \begin{array}{c}\hfill HOMA1-IR=\frac{\left[ fasting\  glucose\right] \times \left[ fasting\  insulin\right]}{22.5}\hfill \\ {}\hfill WBISI=\frac{10000}{\sqrt{\left[ fasting\  glucose\right]\times \left[ fasting\  insulin\right] \times \left[ mean\  OGTT\  glucose\right]\times \left[ mean\  OGTT\  insulin\ \right]}}\hfill \end{array} $$

Height, body weight and blood pressure were measured [27], blood samples for measurement of liver enzymes (AST, ALT, γ-GT) were collected, and transabdominal ultrasonography was performed as described below.

The participants included from the Tromsø Study follow-up were recruited from three groups: participants with either AST, ALTor γ-glutamyl transferase >2× Upper Limit of Normal, participants with values between ULN and 2× ULN, and a selection of participants with normal values, matched for sex and age of the first group (enzyme values >2× ULN).

The group with liver enzyme levels >2× ULN was followed up during the first few months after the Tromsø Study visits in 2008. The two other groups were followed up during 2013/2014. The same variables were recorded for all three groups: height, body weight, liver enzyme levels, and fasting blood glucose, serum insulin, and fasting triglyceride levels. Transabdominal ultrasonography was performed with measurement of HRI. Blood pressure was also measured from the Tromsø study visits.

Transabdominal ultrasonography was performed using a Hitachi EUB-6500 HW apparatus with a 5 MHz convex EUP-C524 transducer (Hitachi Medical Corporation, Tokyo, Japan). Hepatic and renal parenchymal echogenic density on a grayscale (values 0–255) was recorded with the built-in histogram function. An average of three repeated measurements was used to calculate HRI by the formula:

$$ HRI = \frac{mean\  liver\  echogenicity}{mean\  kidney\  echogenicity} $$

Values below 1.0 were corrected to 1.0. Steatosis was classified as mild (HRI 1.05–1.24), moderate (HRI 1.25–1.64) or severe (HRI ≥1.65) [26].

All statistical analyses were carried out using IBM SPSS Statistics, version 21. Receiver Operating Characteristic analyses (ROC) were performed, detecting IR at HOMA1-IR values >2.3 and >2.5 in both inclusion groups combined.

The cut-off value HOMA1-IR >2.3 has previously been described [24]. Other studies have previously used a value of HOMA1-IR >2.7. In this study, the cut-off value is set to 2.5 due to our relatively small study sample, with few observations of HOMA1-IR >2.7.

Subgroups of the participants with BMI ≥30 (*n* = 46) and BMI ≥35 (*n* = 27) were analysed separately.

## Results

In total, 90 participants were included (20 men and 70 women), of which 22 participants were included from the Centre of Obesity and 68 participants from the Sixth Tromsø Study population as shown in Fig. 1. Baseline characteristics are shown in Table 1.

**Table 1:** Baseline characteristics of the 90 study participants

	Inclusion groups					
	Centre of Obesity			Sixth Tromsø Study		
	N	Median (SD)	Range	N	Median (SD)	Range
Age, years	22	43.0 (12.76)	21–69	68	66.0 (10.84)	32–82
Height, cm	22	167.5 (6.87)	156–179	68	166.0 (9.08)	141–189
Weight, kg	22	113.0 (16.13)	83.5–148.0	68	81.3 (15.51)	50.6–123.5
Systolic BP, mmHg	22	126.0 (11.36)	112–159	68	137.0 (22.07)	96–213
Diastolic BP, mmHg	22	74.0 (8.16)	62–94	68	78.0 (8.42)	50–102
BMI, kg/m^2^	22	41.8 (5.66)	31.8–52.7	68	28.0 (5.35)	19.3–45.6
HOMA1-IR	22	2.5 (1.75)	0.8–6.5	65	0.8 (3.18)	0.2–25.5
WBISI	22	0.3 (0.77)	0.03–2.9	0	-	-
AST, U/L	22	18.0 (9.32)	12.0–53.0	66	29.5 (1.23)	14.0–71.0
ALT, U/L	4	38.5 (6.70)	29.0–45.0	66	39.0 (20.36)	14.0–102.0
γ-GT, U/L	21	29.0 (45.72)	13.0–198.0	65	82.0 (82.74)	14.0–398.0
ALP, U/L	4	88.0 (9.22)	80.0–99.0	66	81.0 (38.96)	36.0–322.0

*SD* Standard Deviation, *BP* Blood Pressure, *BMI* Body Mass Index, *HOMA1-IR* Homeostasis Model Assessment of Insulin Resistance 1, *WBISI* Whole Body Insulin Sensitivity Index, *AST* Aspartate Aminotransferase, *ALT* Alanine Aminotransferase, *γ-GT* Gamma-Glutamyl Transferase, *ALP* Alkaline Phosphatase

**Fig. 2:**
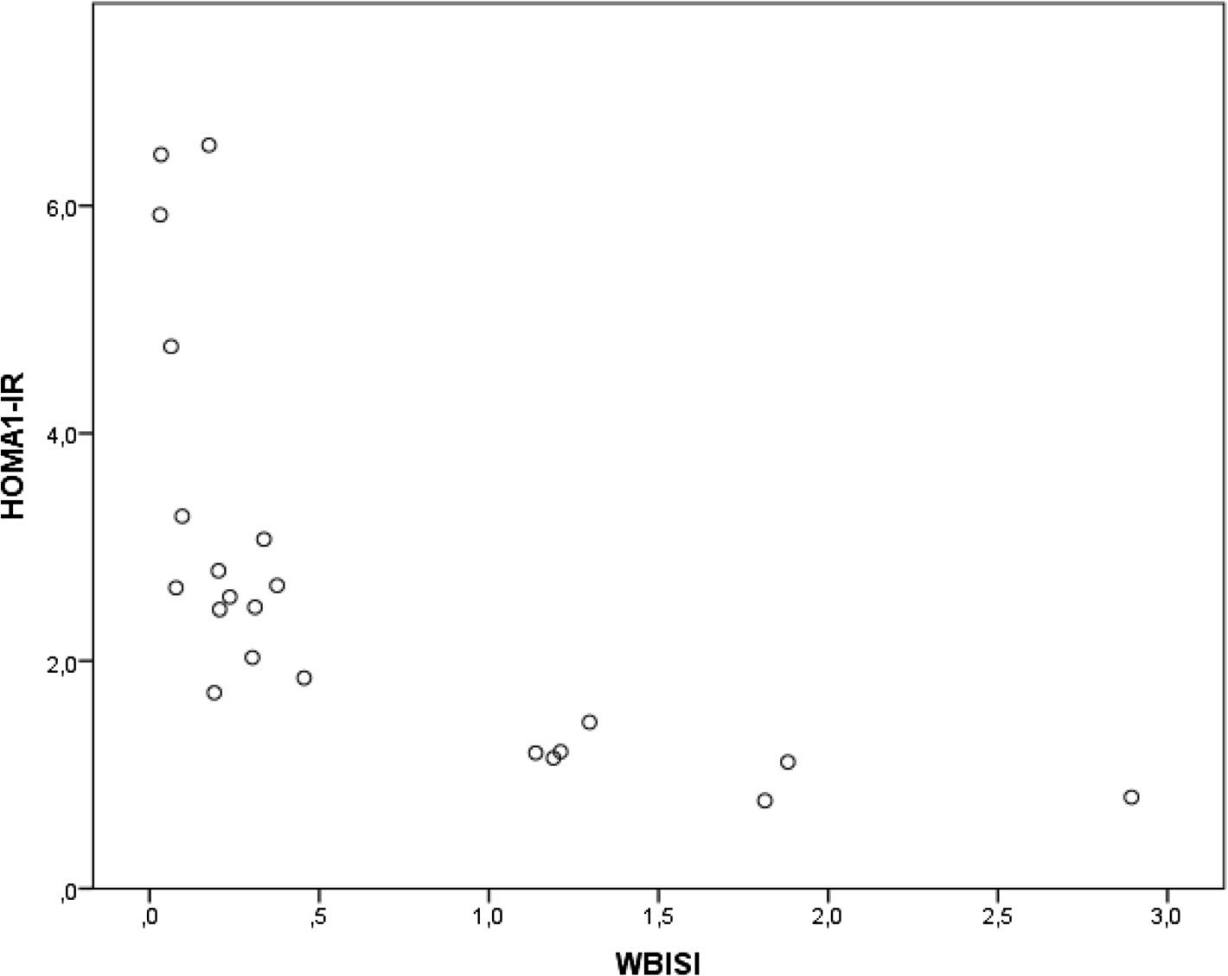
Correlation between WBISI and HOMA-IR for 22 overweight or obese participants. Spearman’s corr. 0.862 (*p* <0,001). Spearman Correlation: −0.922 (*p* <0.001) (two-tailed analysis)

For the participants included from the Centre of Obesity, we calculated both HOMA1-IR and WBISI, in order to verify the reliability of HOMA1-IR in our data set. The correlation between HOMA1-IR and WBISI is shown in Fig. 2.

There were five missing values for either HOMA1-IR or HRI in the dataset. Hence, we performed the ROC analysis with 85 participants (all cases). Sensitivity and specificity values for different HRI levels for detecting IR measured by HOMA1-IR are shown in Table 2. We made the choice of cut-off values for HRI based on a trade-off between sensitivity and specificity of the test. The test properties and likelihood ratios derived from the ROC analysis by 2 × 2 tables are shown in Table 3.

**Table 2:** Test properties for different levels of hepatorenal index for the prediction of insulin resistance (HOMA1-IR)

**HOMA-IR >2.3**						
	All participants (n = 85) (n = 21 with HOMA-IR >2.3)		BMI ≥30 (n = 45) (n = 18 with HOMA-IR >2.3)		BMI ≥35 (n = 27) (n = 15 with HOMA-IR >2.3)	
HRI cut-off	1.17	1.42	1.17	1.42	1.17	1.42
Sensitivity	0.90 (.71; .97)	0.33 (.17; .55)	0.94 (.74; .99)	0.33 (.16; .56)	0.93 (.70; .99)	0.27 (.11; .52)
Specificity	0.70 (.58; .80)	0.94 (.85; .98)	0.70 (.52; .84)	0.96 (.82; .99)	0.75 (.47; .91)	0.92 (.65; 99)
LR +	3.05 (2.04; 4.56)	5.33 (1.73; 16.4)	3.19 (1.76; 5.76)	9.0 (1.18; 68.6)	3.73 (1.39; 10.0)	3.20 (.41; 25.0)
LR -	0.14 (.04; .51)	0.71 (.52; .97)	0.08 (.01; .54)	0.69 (.50; .97)	0.09 (.01; .61)	0.80 (.56; 1.14)
**HOMA-IR >2.5**						
	All participants (n = 85) (n = 17 with HOMA-IR >2.5)		BMI ≥30 (n = 45) (n = 14 with HOMA-IR >2.5)		BMI ≥35 (n = 27) (n = 12 with HOMA-IR >2.5)	
HRI cut-off	1.17	1.42	1.17	1.42	1.17	1.42
Sensitivity	0.88 (.66; .97)	0.29 (.13; .53)	0.93 (.69; .99)	0.29 (.12; .55)	0.92 (.56; .99)	0.18 (.05; .48)
Specificity	0.66 (.54; .76)	0.94 (.82; .94)	0.61 (.44; 76)	0.90 (.75; 97)	0.60 (.36; .80)	0.81 (.57; .93)
LR +	2.61 (1.79; 3.80)	4.85 (1.15; 9.63)	2.40 (1.51; 3.82)	2.95 (.76; 11.4)	2.29 (1.21; 4.36)	0.97 (.19; 4.88)
LR -	0.18 (.05; .66)	0.75 (.57; 1.06)	0.12 (.02; 79)	0.79 (.56; 1.12)	0.14 (.02; 95)	1.01 (.70; 1.45)

Test properties and likelihood ratios (LR ±) for different cut-off values of hepatorenal index (HRI) for predicting different levels of insulin resistance, defined by Homeostasis Model Assessment of Insulin Resistance (HOMA1-IR) >2.3 and 2.5, respectively. BMI subgroups are analysed specifically

**Table 3:** 2 × 2 tables for detecting insulin resistance (HOMA1-IR >2.3) by mild hepatic steatosis (HRI ≥1.17)

**All cases**				
		HOMA1-IR		
		>2.3	≤2.3	Total
HRI	≥1.17	19	19	38
	<1.17	2	45	47
	Total	21	64	85
**BMI ≥ 30**				
		HOMA1-IR		
		>2.3	≤2.3	Total
HRI	≥1.17	17	8	25
	<1.17	1	19	20
	Total	18	27	45
**BMI ≥ 35**				
		HOMA1-IR		
		>2.3	≤2.3	Total
HRI	≥1.17	14	3	17
	<1.17	1	9	10
	Total	15	12	27

Overall, 45 % (*n* = 38) of all subjects had HRI ≥1.17, while 13 % (n = 11) had HRI ≥1.42.

**Fig. 3:**
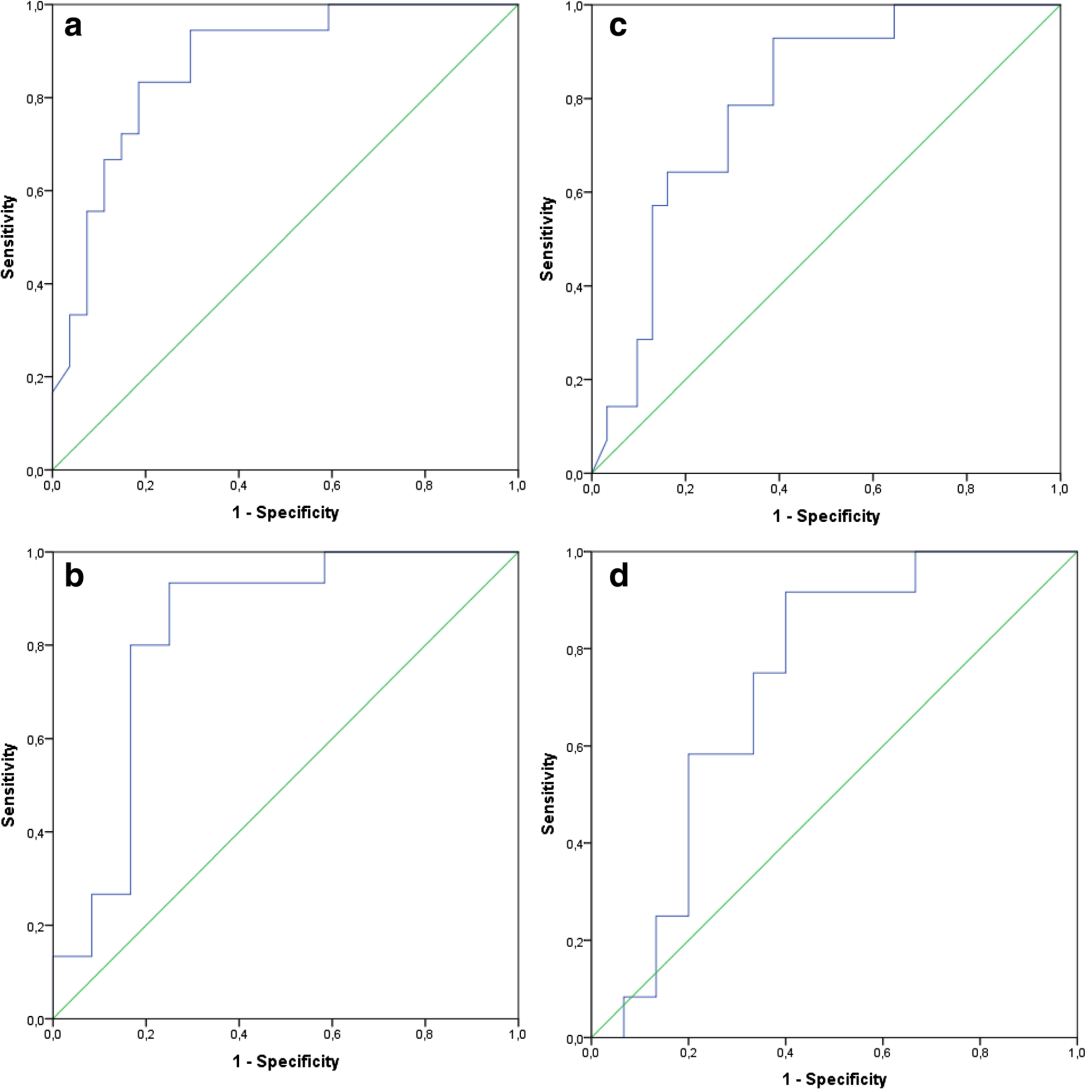
**a** ROC curve of HOMA-IR >2.3 by HRI in participants with BMI ≥30. **b** ROC curve of HOMA-IR >2.3 by HRI in participants with BMI ≥35. **c** ROC curve of HOMA-IR >2.5 by HRI in participants with BMI ≥30. **d** ROC curve of HOMA-IR >2.5 by HRI in participants with BMI ≥ 35

The test has a high sensitivity and a relatively low specificity for HRI values corresponding to mild hepatic steatosis (HRI = 1.17), and a low sensitivity with a high specificity for HRI values corresponding to moderate hepatic steatosis (HRI = 1.42). The corresponding ROC curves are shown in Fig. 3a–d.

## Discussion

Our results show that the detection and quantification of hepatic steatosis by the measurement of abnormal hepatorenal index values is feasible as a screening tool for the detection of insulin resistance. However, HRI values corresponding to mild steatosis showed a high sensitivity but a relatively low specificity. On the other hand, HRI values corresponding to moderate steatosis had a low sensitivity but a high specificity. Therefore, one must weigh the importance of finding patients with IR against the inconvenience of having many false positive results from the test.

As far as we know, the relationship between an abnormal HRI and abnormal HOMA1-IR values has not been investigated before, despite the invariable association between NAFLD, obesity and MetS [5]. In light of this association, having good screening methods for detecting early signs of MetS, especially in obese patients, is of great importance. Also in normal-weight subjects at risk, a good screening tool is useful. In normal-weight and non-diabetic individuals the correlation between the components of MetS, visceral fat accumulation and IR has shown to correlate with the severity of NAFLD and all the different components of MetS are strongly associated with NAFLD [8, 28]. Transabdominal ultrasonography (US) is already in use for bedside diagnostics of NAFLD, but subjective evaluation of steatosis characteristics in US is susceptible to inter and intra observer variability, especially for mild and moderate steatosis [25]. Our results indicate that the use of HRI will improve the test properties of US in the diagnostics of MetS.

IR in obese individuals is crucial for the risk of further development of MetS, and the presence of hepatic steatosis and IR is closely linked. Although the HOMA1-IR is relatively simple to calculate both in general practice and in specialist care, it tends not to be in mind of the clinician, unless there are other factors suggesting an underlying IR.

A previous study by Geloneze et al. has shown that the optimal cut-off value of HOMA1-IR is 2.3 for detecting insulin resistance and MetS (sensitivity 77 % and specificity 67 %) [24]. The updated HOMA2-IR is more accurate, correcting for feedback relationships between insulin resistances in different organs [24]. The optimal cut-off value for HOMA2-IR is 1.4, (sensitivity 79 % and specificity 62 %). However, HOMA2-IR has a limited range of reliable values, and calculating the index is much more complicated [24].

A second method for assessing IR is the Whole Body Insulin Sensitivity Index (WBISI), which adds predictive value to HOMA-IR for assessing risk of IR, but is more inconvenient to use in clinical practice because of the need of an Oral Glucose Tolerance Test (OGTT), measuring postprandial blood glucose and serum insulin values in addition to fasting levels [23, 29]. We have demonstrated a good correlation between WBISI and HOMA1-IR, and thus the usefulness of HOMA1-IR in this study.

Our cut-off value of HOMA1-IR >2.3 was set in accordance to the study by Geloneze et al. [24]. Our results show that using HRI as a screening test for detecting IR is possible, but it should only be used in groups where the prevalence of IR is high, that is, in people with BMI >30. In this group, mild steatosis (HRI ≥1.17) diagnosed by ultrasonography, detected 94 % of patients with a HOMA1-IR of more than 2.3. However, the specificity of the test is low (70 %). Therefore, this test will identify patients with high risk of having IR.

We also calculated test properties for HOMA-IR level 2.5, because the clinical significance of the HOMA1-IR limit may vary, depending on whether one is interested in a high sensitivity or a high specificity of HOMA1-IR in diagnosing patients with true IR [24]. A limit of 2.5 gives a more clinically applicable HOMA-IR value, but with a higher number of false negative results.

The gold standard for diagnosing hepatic steatosis is by liver biopsy [30], which also is the only way of diagnosing the presence of steatohepatitis. Liver biopsy, however, remains an invasive procedure with a risk of complications, and the need for biopsy in the diagnosis of NAFLD is much debated [31]. Ultrasonography, being a risk-free non-invasive procedure, that is simple to perform, would be a good choice for clinical screening.

In a previous study of the test properties of HRI performed during abdominal ultrasonography, it was shown that a HRI cut-off value of 1.49 has a sensitivity of 100 % and a specificity of 91 % for detecting a 5 % steatosis, diagnosed by liver biopsy [26]. Our results show that a HRI cut-off at 1.42 will have a specificity of 96 % in the obese group with BMI ≥30 for having HOMA1-IR >2.3. A HRI value ≥1.49 will therefore be diagnostic, for both having an actual hepatic steatosis, and also actually having a HOMA1- IR of more than 2.3, and thus having insulin resistance.

Although a lower HRI cut-off level of ≥1.17 will give many false positive results, this is acceptable in a screening test, since the verification of IR is relatively simple through calculating HOMA1-IR.

One of the strengths of this study is that the study population is similar to the patient group that will be relevant for this screening, both in regards to overweight and obesity, as well as pathological liver enzyme levels. An extrapolation to the general population is, however, not possible.

We did not perform liver biopsy to confirm the results of the ultrasound examination. Therefore, one cannot say that participants with HRI ≥1.49 have hepatic steatosis. The correlation between the actual steatosis and IR is beside the scope of this study, since this correlation is generally accepted [13].

One of the weaknesses of using HRI as a means of assessing steatosis is its variability. There is a certain degree of both inter- and intra-observer variability, but the results are also dependent on the type of ultrasound equipment used. An example of this is one of the features available in later ultrasonography equipment models, i.e. the option to highlight the liver tissue above other tissues. Thus, the actual HRI values in different studies may not be directly comparable as a result, and one needs to be aware of this as a source of bias when choosing this method.

The participants were not examined for other chronic liver diseases, apart from a medical history. This is a possible source of bias, since the presence of liver cirrhosis could influence the HRI measurements.

The small sample size is a weakness of this study, particularly in the subgroup of morbid obese participants (BMI ≥ 35), where only 12 participants had HOMA1-IR >2.3.

Because of this, the results of our study need confirmation by further studies, with more participants included.

## Conclusion

The detection of hepatic steatosis by transabdominal ultrasound, and the quantification of steatosis by measurement of the hepatorenal index, is a feasible screening tool for stratifying patients with regards to risk of having insulin resistance: patients with low risk of IR (HRI <1.17), patients at risk of having IR (HRI 1.17–1.41), and patients with likely IR (HRI ≥1.42). The test should primarily be used in obese subjects.
